# The Essentiality of Reporting Hardy-Weinberg Equilibrium Calculations in Population-Based Genetic Association Studies

**DOI:** 10.22074/cellj.2016.3711

**Published:** 2015-07-11

**Authors:** Atefeh Namipashaki, Zahra Razaghi-Moghadam, Naser Ansari-Pour

**Affiliations:** 1 Department of Medical Biotechnology, School of Advanced Technologies in Medicine, Tehran University of Medical Sciences, Tehran, Iran; 2 Faculty of New Sciences and Technology, University of Tehran, Tehran, Iran

**Keywords:** Genetic Association, Hardy-Weinberg Equilibrium, Population Stratification, Polymorphism, Bias

## Abstract

Population-based genetic association studies have proven to be a powerful tool in identifying genes implicated in many complex human diseases that have a huge impact on public health. An essential quality control step in such studies is to undertake Hardy-Weinberg equilibrium (HWE) calculations. Deviations from HWE in the control group may reflect important problems including selection bias, population stratification and genotyping errors. If HWE is violated, the inferences of these studies may thus be biased. We therefore aimed to examine the extent to which HWE calculations are reported in genetic association studies published in Cell Journal_(Yakhteh)_(Cell J). Using keywords pertaining to genetic association studies, eleven relevant articles were identified of which ten provided full genotypic data. The genotype distribution of 16 single nucleotide polymorphisms (SNPs) was re-analyzed for HWE by using three different methods where appropriate. HWE was not reported in 60% of all articles investigated. Among those reporting, only one article provided calculations correctly and in detail. Therefore, 90% of articles analyzed failed to provide sufficient HWE data. Interestingly, three articles had significant HWE deviation in their control groups of which one highly deviated from HWE expectations (P= 9.8×10^-12^). We thus show that HWE calculations are under-reported in genetic association studies published in this journal. Furthermore, the conclusions of the three studies showing significant HWE in their control groups should be treated cautiously as they may be potentially misleading. We therefore recommend that reporting of detailed HWE calculations should become mandatory for such studies in the future.

## Introduction

Identification of genes underlying human traits including diseases is crucial to our understanding of their etiology and is an important prerequisite for clinical diagnostics and prophylaxis (1). One common strategy in identifying such genes has been the candidate gene association approach (2). Although this approach requires knowledge for prioritizing genes for screening, it benefits from simplicity in design and has thus attracted the attention of many investigators. According to the PubMed database, over 35,000 papers have been published which contain the keywords "genetic polymorphism" and "disease". Interestingly, in the post-genomic era, the candidate gene approach has not only lost popularity, it is still pursued for unraveling the genetics of many complex diseases hitherto [for a recent example in cancer research see Ruark et al. (3)]. In this approach, case-control analysis, compared with familial transmission disequilibrium test (TDT) (4), has been by far the most commonly employed design (5). This design aims to detect loci, at the population level, for which allelic or genotypic status correlates with disease outcome by comparing unrelated cases and controls. In practical terms, it is relatively easy to implement. For instance, recruiting large number of unrelated participants is relatively easier than family-based sampling and also results in increased statistical power (6). However, with this comes certain drawbacks of which subject selection in creating a control group, to compare with the case group, is quite challenging (7). The control group should represent the general population of the region where patients emanate from and be free of the disease present in case-group individuals. Clinical assessment of the control group can somewhat eliminate the possibility of disease presence, however, fulfilling the former criterion is not easily established and may result in biased inferences. Moreover, population stratification can also lead to spurious associations (8) when the control group represents more than one ethnic group with varying allele frequencies. One way to address both representativeness and homogeneity (i.e. lack of significant population stratification) of the control group is to ensure that observed genotypic frequencies are compatible with Hardy-Weinberg equilibrium (HWE) predictions (8, 9). The Hardy- Weinberg law, which is the basis of population genetics, states, in part, that in a large randommating population at equilibrium (i.e. no selection, migration or genetic drift), genotype frequencies are functions of allele frequencies and the former can be predicted from the latter. Therefore significant deviations from HWE predictions could be a reflection of violation of HWE assumptions in the general population but it can also stem from other sources such as population stratification (8, 10) and genotyping errors (10-13). This bias if unchecked could result in biased conclusions (i.e. accepting or refuting an association while it is otherwise) (14). Typically, HWE does not need to hold for the case-group since they are a non-random selection of individuals based on a phenotype of interest (i.e. disease). Furthermore, interestingly HWE deviation has been proposed as a measure of disease association when analyzing the case group *per se* (15-18).

HWE is typically assessed using a Chi-square goodness-of-fit test. However, when genotype frequencies are low (genotype counts below 5), the Chi-square approximation of the test statistic is poor and an exact test should be used as an alternative (19). Recently, Wellek et al. (20) pointed out that these methods test for deviation and do not directly test the alternative hypothesis of compatibility. They also presented a confidence interval (CI)-based test of the ratio ω [a measure of relative excess heterozygosity (REH)] to test HWE compatibility directly.

There is accumulating evidence from multiple surveys (21-24) that HWE calculations are not reported in a considerable subset of population-based genetic association studies in different journals and lack of reporting ranged from 31-80% (23). These surveys also pointed out that this tool has been sometimes misapplied resulting in probable biased conclusions. In this retrospective survey, we examined reporting of HWE compatibility in population-based case-control genetic association studies published in Cell Journal_(Yakhteh)_ (Cell J).

## Results

Sixteen genotype distributions of ten eligible articles were re-analyzed (see Supplementary Online Information for Materials and Methods at www.celljournal.org). Six articles (60%), reporting a total of eight SNPs, failed to report HWE calculations (Table 1). Based on the genotype distributions reported, we identified three SNPs (out of 8) deviating from HWE of which two were in control groups (Studies D and J) and one in a case group (Study G). Of those reporting to have undertaken HWE calculations (40%), two failed to report corresponding pvalues and found it either sufficient to make a general statement (for only one of the SNPs and not both) of HWE fulfillment (Study H) or completely ignored to comment on their HWE findings (Study I). Interestingly, among those two reporting HWE P values, one states that both case and control groups are in HWE, despite a significant deviation in the control group (reanalyzed P=0.005) (Study C). This article also incorrectly states that degrees of freedom (*df*) for a Chi-square based HWE test is two while *df*=1. Correct P values from our re-analysis of genotypic distributions plus further details are given in table 1.

**Table 1:** Summary of analyses undertaken on genotype distributions of 16 SNPs reported in genetic association studies included in this study


Study	Article^a,b^	Gene(Polymorphism)	Group	N	Genotype (N)			P value(Re-analysis)^c^	P value(Article)	REH(95% CI)^f^	HWE Reported
					AA	AB	BB				

A	Dastgerdi andSadeghi (2009)	TP53(R72P)	Case	144	65	61	18	0.534	-	0.892 (0.621-1.281)	No
			Control	-	-	-	-	-	-	NA	-
B	Bahadoriet al. (2010)	PTPRZ1(rs13241278)	Case	140	46	72	22	0.485	0.5	1.132(0.803-1.595)	Yes
			Control	165	65	72	28	0.327	0.8	0.844(0.613-1.162)	Yes
		PTPRZ1(SNPrs2693657)	Case	140	46	71	23	0.607	0.8	1.091(0.776-1.536)	Yes
			Control	165	49	82	34	1.000	0.99	1.004(0.738-1.366)	Yes
C	Shariatiet al. (2011)	NRG1(SNP8N-RG241930)	Case	95	8	36	51	0.798	(χ2=0.07,df=2, P≤ 0.1)	0.891(0.543-1.463)	Yesbut incorrect
			Control	95	10	60	25	0.005	(χ2=0.12,df=2, P≤0.1)	1.897(1.215-2.962)	Yesbut incorrect
D	Azadeh Sayadet al. (2012)	LPL(Intronic HindIII)	Case	100	58	40	2	0.145	-	1.857(0.86-4.01)	No
			Control	100	44	52	4	0.030	-	1.96(1.098-3.499)	No
E	Pouresmailiet al. (2013)	VDRrs1544410	Case	64	14	33	17	0.797	-	1.07(0.654-1.748)	No
			Control	82	13	33	36	0.330	-	0.763(0.479-1.215)	No
F	Aida Sayadet al. (2013)	IL-2(-475 IL-2)	Case	100	96	4	0	1.000	-	NA	No
			Control	100	100	0	0	NA^d^	-	NA	No
		IL-2(-631 IL-2)	Case	100	98	2	0	1.000	-	NA	No
			Control	100	100	0	0	NA	-	NA	No
G	Pirahmadiet al. (2013)	TLR4(D299G)	Case	350	303	42	5	0.017	-	0.54(0.316-0.922)	No
			Control	350	315	35	0	1.000	-	NA	No
		TLR4	Case	350	296	54	0	0.246	-	NA	No
		(T399I)	Control	350	294	56	0	0.148	-	NA	No
H	Zamaniet al. (2014)	CD14(1359G/T)	Case	100	60	33	7	0.403	-	0.805(0.479-1.353)	Yes
			Control	100	63	32	5	0.766	-	0.901(0.509-1.598)	Yes
		CTLA4(49A/G)	Case	100	61	35	4	1.000	-	1.091(0.776-1.536)	Yes
			Control	100	58	36	6	1.000	-	1.004(0.738-1.366)	Yes
I	TaghizadehMortezaeeet al. (2014)	ESR1(351A/G)	Case	276	93	128	55	0.385	-	0.895(0.704-1.138)	Yes
			Control	157	55	77	25	0.738	-	1.038(0.75-1.437)	Yes
		ESR1(397T/C)	Case	276	78	133	65	0.635	-	0.934(0.737-1.183)	Yes
			Control	157	50	74	33	0.630	-	0.911(0.664-1.25)	Yes
		CYP1A1(I462V)	Case	276	241	35	0	0.611	-	NA	Yes
			Control	157	144	13	0	1.000	-	NA	Yes
J	Motovali-Bashiet al. (2014)	XPD(K751Q)	Case	288	80	144	64	1.000	-	1.006(0.798-1.269)	No
			Control	352	112	112	128	9.8×10^-12e^	-	0.468(0.374 -0.585)	No


^a^; Articles are sorted chronologically and those reporting a significant association are shown in bold type, ^b^; Full details of these articles are given in Appendix 1 of the Supplementary Online Information at www.celljournal.org, ^c^; Significant P values are shown in bold type, ^d^; Not applicable, ^e^; Since this P value approached zero using the Chi-square-based test, HWE exact test was used to obtain the exact P value and ^f^; REH value is reported as ‘NA’ when any genotype count is zero since ω can only take non-zero values. REH CI not containing zero are shown in bold type.

## Discussion

The significance of HWE testing in population-based genetic association studies is immense especially when analyzing the control group (21-24). This is because an important assumption underlying these studies is that the control group is a representative sample of the population under investigation. Another assumption in such studies is that individuals of both case and control groups belong to the same single large random-mating population (25). This in effect assumes that there is a lack of significant population stratification. Therefore, studies that fail to analyze or report HWE, are susceptible to biased inferences and misleading conclusions. In this survey, we have shown that 90% of the articles analyzed failed to report their HWE calculations correctly or in detail. Study B is the only one reporting HWE analysis in full. Although they correctly report lack of deviation for both SNPs in both cases and controls, their P values are not identical to those obtained by us. This discrepancy may be attributable to the difference of methods implemented in software used (R vs. SPSS) to calculate HWE P values.

Studies C, D and J overlooked the deviation from HWE in their control groups. It is essential that the control group fulfils HWE expectations. Consistent with the results of goodness-of-fit test P values, the three SNPs tested for association showed 95% CI of REH above 1, thus confirming HWE incompatibility (Table 1). Interestingly, all three articles report significant genetic associations with disease. In specific, Study C found a significant over-representation of GG homozygotes among schizophrenia patients at SNP8NRG241930 in *NRG1* (P<0.001). However, deviation from HWE in controls was also significant (P=0.005) with a relatively high excess of heterozygotes (F=-0.295). Given that control individuals were sampled from South West Iran, this excess heterozygosity could be a reflection of an isolate-breaking effect (i.e. the mixing of two previously isolated populations) (26) in that region. It would be interesting to speculate that this effect is caused by the mixing of two major ethnicities residing in that area (i.e. Arab and Fars). In Study D, an association with borderline significance was found between the HindIII polymorphism in LPL and late-onset Alzheimer’s disease (P=0.048). We found a significant HWE deviation in the control group (P=0.03) with considerable excess heterozygosity (F=-0.238). Although no detail is given on the geographic region of sampling, this pattern may represent outbreeding in the population that they emanate from. Study J reported a border-line association between the heterozygote state at a missense SNP (K751Q) in *XPD* and lung cancer risk (P=0.047) but not for the overall genotype distribution. However, we obtained a highly significant HWE deviation (P=9.8×10^-12^) in the control group. If we assume that the observed heterozygosity is true, the coefficient of inbreeding is relatively high (F=0.36) thus indicating that control samples are either not a set of unrelated individuals or population stratification exists in the source population. Since population stratification always decreases the number of heterozygotes (27), it is likely that this deficit of heterozygotes is a reflection of this. Inbreeding in the population could also be the source of this, however, since individuals were randomly sampled from those referring to a hospital for regular check-ups in Isfahan (a metropolitan city in Central Iran with a relatively large population), it is more likely that population stratification is at play. Although genotyping error has been suggested to be a source of HWE deviation (12, 13), this seems not to be a probable reason for this observation given that the case group genotypic distribution follows HWE (P=1, F ≈ 0) while this is not a must for case groups.

The conclusions made by these three studies thus need to be dealt with caution since the observed HWE deviation in the control groups creates bias creates bias in the result of the associations reported (21-23). It is thus worth re-assessing these associations using new sets of controls which follow HWE expectations to see whether these associations remain significant. For instance, assuming the same allele frequencies, had the genotype frequencies followed HWE in the control group in Study C, the association would have remained significant albeit with a lower significance level (re-analyzed association P=0.012).

On a contrary note, after working out genotypic distributions for the two SNPs tested in TLR4 in Study G (Table 1), HWE deviation was observed only in the case group for SNP D299G (P=0.017). This incompatibility may be a signal of disease association (16). Interestingly, when we assessed association between each SNP and malaria infection risk (not undertaken by the authors), SNP D299G reached significance level (P=0.046). Based on these two corroborating observations, it is therefore plausible to suggest that this missense SNP is a malariaassociated disease marker but went unnoticed by the authors. This finding has practical consequence for future population-based association studies. It shows that testing HWE not only identifies SNPs to be discarded from such studies (due to HWE deviation) and acts as a key quality control step (11), it can also help detect less straightforward associations.

## Conclusion

We show that test of HWE is an underused tool in Cell J articles reporting genetic association studies with three studies resulting in probable biased associations and one study overlooking a likely association. It is therefore recommended that reporting of detailed HWE calculations should become mandatory for such articles in the future. On a more general note, it is our belief that this journal should endorse STREGA (28) by asking authors to adhere to its recommendations. This would undoubtedly improve reporting of genetic association studies as well as help researchers to evaluate such studies more conveniently.

## Supplementary PDF


