# Supplementary material for: The Essentiality of Reporting Hardy-Weinberg Equilibrium Calculations in Population-Based Genetic Association Studies
Source: Cell J. 2015 Jul 11;17(2):187–92. doi: 10.22074/cellj.2016.3711 (PMC4503832; doi:10.22074/cellj.2016.3711)
Supplement: Supplementary file 1 [file Cell-J-17-187-s01.pdf]

CELL JOURNAL (Yakhteh)

www.celljournal.org

Supplementary Information for

The Essentiality of Reporting Hardy-Weinberg Equilibrium Calculations in Population-Based Genetic Association Studies

Atefeh Namipashaki, M.Sc.[1], Zahra Razaghi-Moghadam, Ph.D.[2], Naser Ansari-Pour, Ph.D.[2]*

1. Department of Medical Biotechnology, School of Advanced Technologies in Medicine, Tehran University of Medical Sciences, Tehran, Iran
2. Faculty of New Sciences and Technology, University of Tehran, Tehran, Iran

***To whom correspondence should be addressed.**
Email: n.ansaripour@ut.ac.ir
Published Summer 2015 at Cell J

This PDF file includes:

Materials and Methods
Appendix 1

Materials and Methods

Article selection

Using the archive database of Cell Journal$_{\text{(Yakhteh)}}$ (Cell J), we scoured through articles using the keywords "polymorphism", "association", "SNP" and "genotype". We identified eleven articles reporting association studies of which one did not provide genotypic data in full and was thus removed from further analysis (1). Also, when combined genotypic frequencies were available for more than one SNP, raw frequencies for each SNP were obtained by analyzing their tabulated data.

Data analysis

Ten articles reporting detailed genotypic data were reviewed (see Appendix 1). One study did not conform to the typical case-control association study by not having a control group. However, since identifying deviation in the case group is also meaningful, we did not exclude this study from analysis. A total of 16 diallelic single nucleotide polymorphisms (SNPs) were reported for which HWE calculations were undertaken using Chi-square goodness-of-fit test (R function *HWE.chisq*) or an exact test (R function *HWE.exact*) when any genotype count was below 5. Also, relative excess heterozygosity (REH) and its 95% CI, as a direct measure of HWE compatibility, was calculated to compare to the result of goodness-of-fit tests and confirm incompatibility (2). Re-analysis of genetic associations at the allelic level was undertaken using Fisher's exact test. These analyses were implemented in the R environment (v 3.0.2) (3) using the 'genetics' and 'stats' packages. Furthermore, Wright's inbreeding coefficient (F) (4) was estimated from $F=1-Ho/He$ where Ho and He are the observed and expected heterozygosity for a SNP in a given group. The statistical significance level was set to $P<0.05$.

References

1. Sayad A, Akbari MT, Pajouhi M, Mostafavi F, Kazemnejad A, Zamani M. Investigation the role of gender on the hla-drb1 and -dqb1 association with type 1 diabetes mellitus in iranian patients. Cell J. 2013; 15(2): 108-115.
2. Wellek S, Goddard KA, Ziegler A.A confidence-limit-based approach to the assessment of Hardy-Weinberg equilibrium. Biom J. 2010; 52(2): 253-270.
3. Team RC. R: A language and environment for statistical computing. (R foundation for statistical computing, Vienna, Austria, 2012). 2012.
4. Wright S. Evolution and the genetics of populations. Variability within and among natural populations. Chicago, IL, USA: University of Chicago Press; 1978.

Appendix 1. References with Eligible Data

1. Motovali-Bashi M, Rezaei H, Dehghanian F, Rezaei H. association between xpd (lys751g1n) polymorphism and lung cancer risk: a population-based study in Iran. Cell J. 2014. 16(3): 309-314.
2. Pirahmadi S, Zakeri S, Abouie Mehrizi A. Multiple genotypes of the commonly co-segregating toll-like receptor 4 asp299gly and thr399ile in baluchi malaria patients from iran. Cell J. 2013; 15(2): 182-189.
3. Pouresmaeili F, Jamshidi J, Azargashb E, Samangouee S. Association between vitamin d receptor gene bsmi polymorphism and bone mineral density in a population of 146 Iranian women. Cell J. 2013; 15(1): 75-82.
4. Sayad A, Allameh A, Sayad A, Noruzinia M, Akbari MT, Sarzaeem A ,et al. The association of -475 and -631 interleukin-2 gene polymorphism with multiple sclerosis in Iranian patients. Cell J. 2013; 15(2): 124-129.
5. Sayad A, Noruzinia M, Zamani M, Harirchian MH, Kazemnejad A. Lipoprotein lipase hindiii intronic polymorphism in a subset of Iranian patients with late-onset alzheimer's disease. Cell J. 2012; 14(1): 67-72.
6. Shariati SAM, Behmanesh M, Galehdari H. A study of the association between snp8nrg241930 in the 5´ end of neuroglin 1 gene with schizophrenia in a group of Iranian patients. Cell J. 2011; 13(2): 91-96.
7. Taghizade Mortezaee F, Tabatabaiefar MA, Hashemzadeh Chaleshtori M, Miraj S. Lack of association between esr1 and cyp1a1 gene polymorphisms and susceptibility to uterine leiomyoma in female patients of Iranian descent. Cell J. 2014; 16(2): 225-230.
8. Zamani M, Karami F, Shirvani F, Kia-Lashaki L, Shahbazkhani B. The role of cd14 and ctla4 gene polymorphisms in risk of celiac disease among patients of Iranian ethnicity. Cell J. 2014; 16(2): 171-178.
9. Bahadori Z, Behmanesh M, Sahraian M-A, Heidari M. Association study of rs13241278 and rs2693657 polymorphisms of ptprz1 gene with multiple sclerosis in Iranian population. Yakhteh. 2010; 12(3): 341-348.
10. Dastjerdi MN, Sadeghi HM.TP53 codon 72 heterozygosity may promote microsatellite instability in sporadic colorectal cancer. Yakhteh. 2010; 12(1): 25-32.
